# High-Level Production of Lysine in the Yeast Saccharomyces cerevisiae by Rational Design of Homocitrate Synthase

**DOI:** 10.1128/AEM.00600-21

**Published:** 2021-07-13

**Authors:** Shota Isogai, Tomonori Matsushita, Hiroyuki Imanishi, Jirasin Koonthongkaew, Yoichi Toyokawa, Akira Nishimura, Xiao Yi, Romas Kazlauskas, Hiroshi Takagi

**Affiliations:** a Division of Biological Science, Graduate School of Science and Technology, Nara Institute of Science and Technology, Ikoma, Nara, Japan; b Department of Biochemistry, Molecular Biology and Biophysics, University of Minnesota, Saint Paul, Minnesota, USA; c The BioTechnology Institute, University of Minnesota, Saint Paul, Minnesota, USA; University of Buenos Aires

**Keywords:** yeast, lysine, homocitrate synthase, *in silico* docking, feedback inhibition, *Saccharomyces cerevisiae*

## Abstract

Homocitrate synthase (HCS) catalyzes the aldol condensation of 2-oxoglutarate (2-OG) and acetyl coenzyme A (AcCoA) to form homocitrate, which is the first enzyme of the lysine biosynthetic pathway in the yeast Saccharomyces cerevisiae. The HCS activity is tightly regulated via feedback inhibition by the end product lysine. Here, we designed a feedback inhibition-insensitive HCS of S. cerevisiae (ScLys20) for high-level production of lysine in yeast cells. *In silico* docking of the substrate 2-OG and the inhibitor lysine to ScLys20 predicted that the substitution of serine with glutamate at position 385 would be more suitable for desensitization of the lysine feedback inhibition than the substitution from serine to phenylalanine in the already known Ser385Phe variant. Enzymatic analysis revealed that the Ser385Glu variant is far more insensitive to feedback inhibition than the Ser385Phe variant. We also found that the lysine contents in yeast cells expressing the Ser385Glu variant were 4.62- and 1.47-fold higher than those of cells expressing the wild-type HCS and Ser385Phe variant, respectively, due to the extreme desensitization to feedback inhibition. In this study, we obtained highly feedback inhibition-insensitive HCS using *in silico* docking and enzymatic analysis. Our results indicate that the rational engineering of HCS for feedback inhibition desensitization by lysine could be useful for constructing new yeast strains with higher lysine productivity.

**IMPORTANCE** A traditional method for screening toxic analogue-resistant mutants has been established for the breeding of microbes that produce high levels of amino acids, including lysine. However, another efficient strategy is required to further improve their productivity. Homocitrate synthase (HCS) catalyzes the first step of lysine biosynthesis in the yeast Saccharomyces cerevisiae, and its activity is subject to feedback inhibition by lysine. Here, *in silico* design of a key enzyme that regulates the biosynthesis of lysine was utilized to increase the productivity of lysine. We designed HCS for the high-level production of lysine in yeast cells by *in silico* docking simulation. The engineered HCS exhibited much less sensitivity to lysine and conferred higher production of lysine than the already known variant obtained by traditional breeding. The combination of *in silico* design and experimental analysis of a key enzyme will contribute to advances in metabolic engineering for the construction of industrial microorganisms.

## INTRODUCTION

Lysine is one of the essential amino acids for humans and protects various organisms from multiple stresses, such as freezing (1), oxidation (2), and combined high-temperature and dryness (3). Mammals cannot derive a sufficient amount of lysine from vegetable proteins because of the low lysine content in vegetable proteins; therefore, industrially produced lysine is widely used as a feed additive to supply sufficient lysine to livestock, leading to good growth, improved quality of meat, and immunity of animals (4–6). Moreover, addition of lysine to cereals has been shown to contribute to human health (7, 8). Lysine is commercially produced from bacteria such as Corynebacterium glutamicum at a rate of about 2 million tons per year (9). Although the lysine productivity of the yeast Saccharomyces cerevisiae is lower than that of bacteria, S. cerevisiae has received attention as a suitable host for food- and pharmaceutical-grade products due to its generally recognized-as-safe (GRAS) status. Therefore, high-level production of lysine in S. cerevisiae could contribute to the development of high-value-added products, such as yeast extract and livestock feed that include large amounts of lysine.

**FIG 1:**
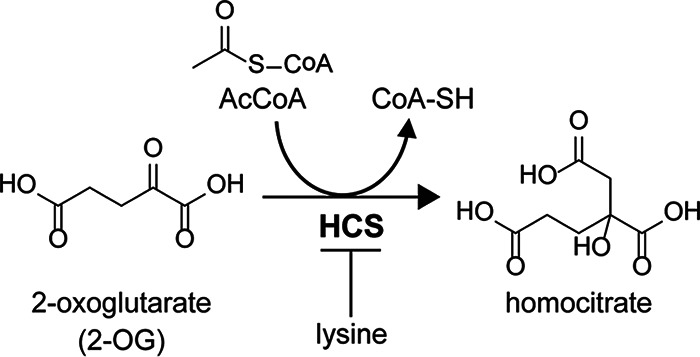
Reaction of HCS. HCS transfers the acetyl group from acetyl coenzyme A (AcCoA) to 2-oxoglutartae (2-OG) to yield homocitrate. The HCS activity is inhibited by lysine in a competitive manner with 2-OG.

Plants and most bacteria biosynthesize lysine via diaminopimelate (DAP) from aspartate; this is known as the DAP pathway (10, 11). On the other hand, fungi, some bacteria, and archaea utilize the α-amino adipate (AAA) pathway for lysine biosynthesis (12–14). The AAA pathway in fungi and yeasts consists of eight enzyme-catalyzed steps. Previous studies of S. cerevisiae and other fungi demonstrated that the AAA pathway is regulated at both genetic and biochemical levels (15). Homocitrate synthase (HCS), which is the first enzyme of the AAA pathway in lysine biosynthesis, transfers the acetyl group of acetyl coenzyme A (AcCoA) to 2-oxoglutarate (2-OG) to yield homocitrate (Fig. 1). This enzymatic reaction is the rate-limiting step in lysine biosynthesis in S. cerevisiae, because the end product, lysine, regulates HCS activity via feedback inhibition (16, 17). Structural and biochemical analyses of HCS of the yeast Schizosaccharomyces pombe and the extreme thermophile Thermus thermophilus demonstrated that the feedback inhibition by lysine is mediated in a competitive manner with substrate 2-OG (18–21). In S. cerevisiae, two paralog HCSs, ScLys20 and ScLys21, are encoded in the genomic DNA, and their enzymatic activities are also inhibited by lysine in the same way as other HCSs. Although these ScHCSs share over 95% amino acid sequence identity, their *K_i_* values for lysine are highly different (550 μM for ScLys20 and 53 μM for ScLys21), indicating that ScLys21 is more sensitive to lysine feedback inhibition than ScLys20 (22–24).

To obtain yeasts that overproduce lysine, a conventional breeding strategy for screening mutants that are resistant to the toxic lysine analog *S*-aminoethyl cysteine (AEC) has traditionally been employed. Several studies reported the isolation of lysine-overproducing mutants derived from the AEC-resistant mutants, and amino acid substitutions were identified in ScLys20 and ScLys21 of these mutants (25, 26); however, this breeding strategy requires a long period of time for mutant isolation and cannot exclude undesirable pseudopositive mutants, leading to a decrease in screening efficiency. Thus, there is a need to develop another strategy that will improve screening efficiency and contribute to an increase in lysine productivity in yeast cells. Recently, a rational design of target enzyme(s) in the metabolic pathway was attempted as a means of enhancing the productivity of useful compounds, including amino acids (27). In this strategy, amino acid substitutions that improve enzymatic properties, such as thermostability (28) and stereoselectivity (29), are designed by *in silico* calculations based on the crystal structure of target and/or homologous enzymes. This rational engineering approach has the potential to identify beneficial variants of target enzymes more efficiently than random mutagenesis combined with screening.

Using *in silico* docking simulation, we predicted here that the substitution of serine with glutamate at position 385 of ScLys20 would lead to the desensitization of feedback inhibition by lysine. *In vitro* and *in vivo* analyses showed that the Ser385Glu variant ScLys20 dramatically reduces the sensitivity to lysine feedback inhibition and markedly increases intracellular lysine content compared with an already-known variant that was obtained by random mutagenesis.

## RESULTS AND DISCUSSION

### Optimization of the amino acid substitution at position 385 of ScLys20 for desensitization to lysine feedback inhibition using *in silico* docking.

**FIG 2:**
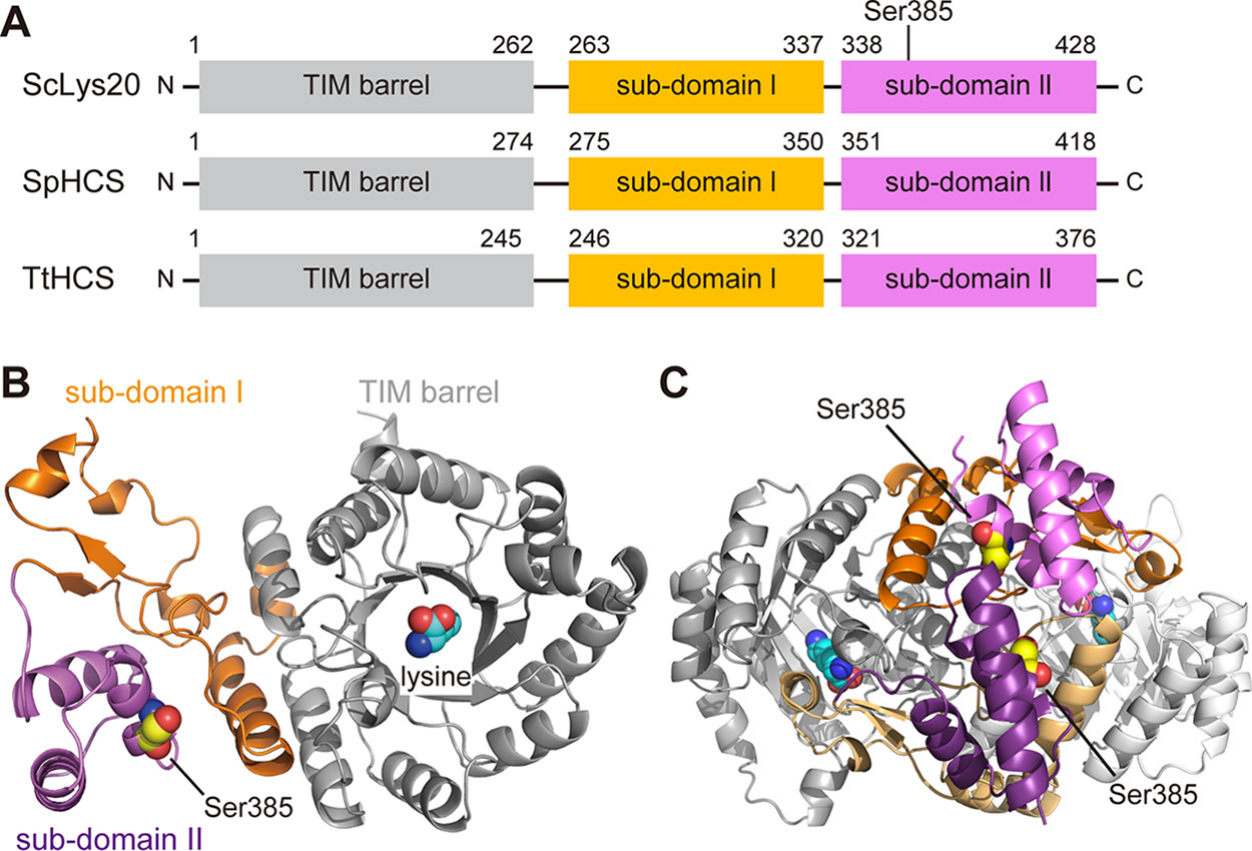
Domain organization of HCSs and homology model of ScLys20. (A) Domain organization of HCSs. ScLys20, Saccharomyces cerevisiae HCS Lys20; SpHCS, Schizosaccharomyces pombe HCS; TtHCS, Thermus thermophilus HCS. (B) Monomer of the ScLys20 homology model (bound with lysine [UniProtKB accession no. P48570]). The whole protein structure is shown by a cartoon model. The TIM barrel domain, subdomain I, and subdomain II are represented in gray, orange, and violet, respectively. Inhibitor lysine bound to the active center and Ser385 are shown in a sphere model in cyan and yellow, respectively. Lysine is predicted to bind the active center consisting of the TIM barrel domain, and Ser385 is located far from the active center in the same monomer. (C) Homodimer of the ScLys20 homology model. Lysine and Ser385 of each monomer are shown in a sphere model in cyan and yellow, respectively. The TIM barrel domain, subdomain I, and subdomain II of the A chain are represented the same as in panel B, while those of B chain are shown as white, bright orange, and purple, respectively. Subdomain I of one monomer is located near the entrance of the active center in the TIM barrel of another monomer.

Structural analysis of the S. pombe HCS (SpHCS) and the T. thermophilus HCS (TtHCS) revealed that the domain organization of HCSs consists of the N-terminal TIM barrel domain with an eight-stranded α/β barrel, which is an active center, and the C-terminal subdomains I and II (Fig. 2A) (18, 19, 21). Comparisons of the amino acid sequences and the three-dimensional homology models suggest that ScLys20 has a domain organization similar to those of SpHCS and TtHCS (Fig. 2A and B). The C-terminal subdomain of SpHCS and TtHCS was shown to play an important role in recognition of the inhibitor lysine (19, 21). A homology model of the ScLys20 homodimer structure suggested that the subdomain I of one monomer is located in the vicinity of the active center of another monomer, as in the case of SpHCS and TtHCS (Fig. 2C). A previous study revealed that multiple amino acid substitutions in the C-terminal subdomain of ScLys20 and ScLys21 decreased the sensitivity to feedback inhibition by lysine (Arg276Lys and Ser385Phe for ScLys20 and Gln366Arg for ScLys21), suggesting that the subdomain of ScHCSs is important for lysine recognition (26).

In S. cerevisiae, among the genes involved in the AAA pathway, overexpression of only *LYS20* conferred an increase in lysine productivity (30). Therefore, ScLys20 is suggested to be a more suitable HCS than ScLys21 for use in engineering to achieve higher production of lysine together with less sensitivity to lysine. We recently isolated a lysine-overproducing mutant derived from the AEC-resistant mutants of S. cerevisiae (unpublished data). This mutant carried an allele of *LYS20*, which encodes the S385F variant of HCS, suggesting the importance of Ser385 for enhancement of the lysine productivity in yeast. Enzymatic analysis of ScLys20 variants indicated that the S385F variant was more subject to lysine feedback inhibition than the R276K variant (26); therefore, replacement of Ser385 by an amino acid residue other than phenylalanine might further reduce feedback inhibition by lysine. Furthermore, no study has described the importance and the function of this serine residue at position 385 for enzymatic activity and feedback inhibition sensitivity of HCSs. Based on this information, we focused on Ser385 as a target residue for the *in silico* design of ScLys20 variants.

**FIG 3:**
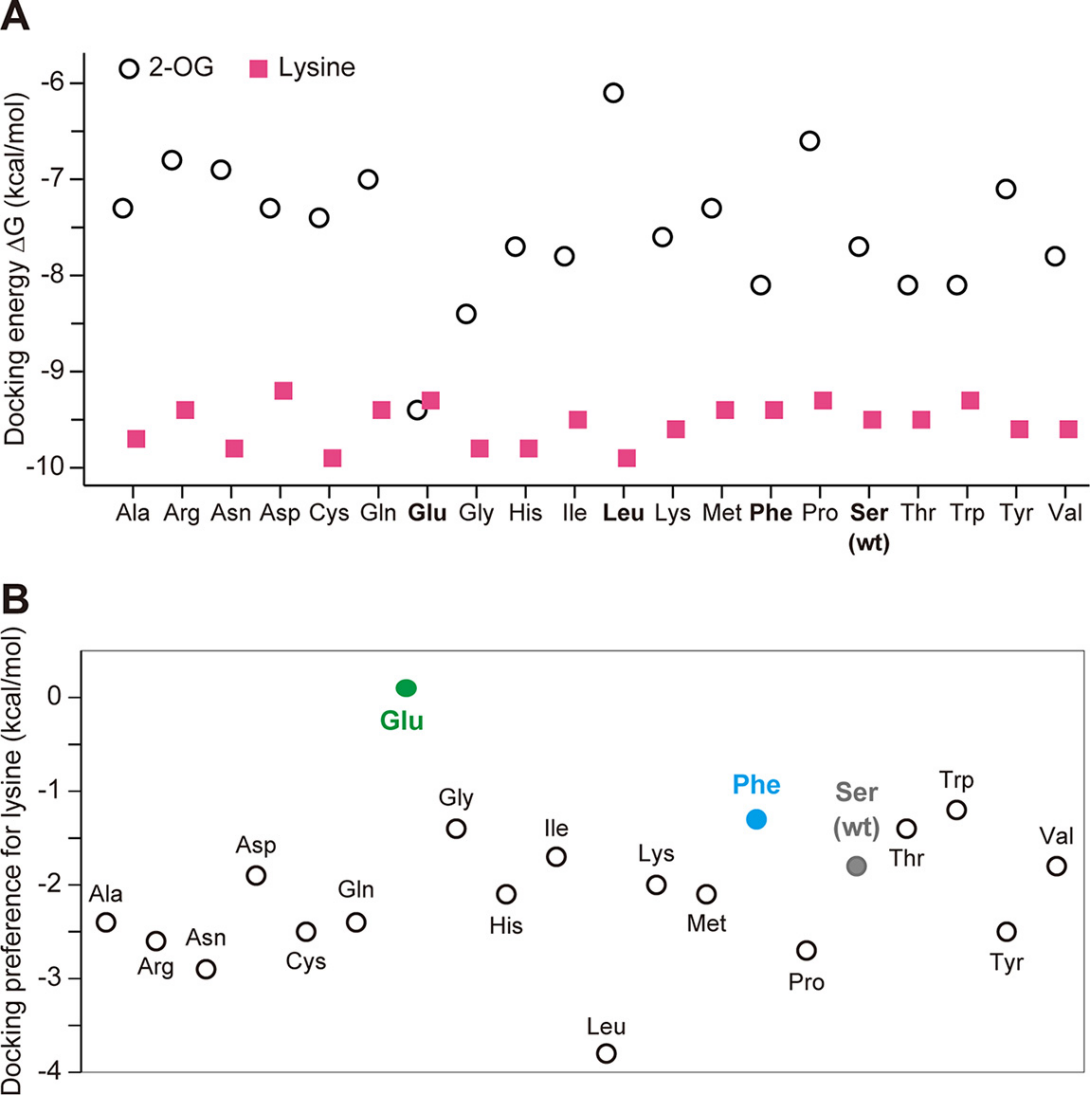
Docking of the substrate 2-OG and the competitive inhibitor lysine to Ser385 variants of ScLys20. (A) The docking energy of lysine (magenta squares) was lower than that for 2-OG (open circles) for all variants, except for Ser385Glu. For this variant, the docking energies of both were similar, suggesting that 2-OG would best compete with lysine for this variant. (B) Differences between the calculated docking energies of 2-OG and lysine. Negative values predict that lysine binds more tightly. The Ser385Glu variant shows the highest relative affinity for 2-OG over lysine (0.1 kcal/mol). The docking was carried out on a homology model of the protein using SwissDock and default parameters. The docking energy of each variant was calculated with binding energies for all poses where the ligand bound within the active site.

Lysine inhibits HCS activity in a competitive manner with 2-OG. Therefore, an amino acid substitution or substitutions that increase the relative affinity for 2-OG over lysine may reduce the sensitivity to lysine. To optimize the amino acid residue at position 385 for desensitization to lysine, the tertiary structures of various Ser385 variants were predicted using a homology model of ScLys20 (bound with lysine) as a template in the SWISS-MODEL repository (31). Subsequently, the binding affinities for the substrate 2-OG and the inhibitor lysine of wild-type ScLys20 (WT-ScLys20) and its variants were calculated using SwissDock (32) (Fig. 3A). The estimated affinity of WT-ScLys20 for 2-OG and lysine indicates that lysine binds more tightly than 2-OG by 1.8 kcal/mol (Fig. 3B, gray circle), or approximately 20-fold more tightly than 2-OG. This estimate agrees with the experimental observation that lysine strongly inhibits HCS activity of ScLys20. The S385F variant of ScLys20 is 100-fold less sensitive to inhibition than the WT enzyme (26). The estimated relative affinity of lysine versus 2-OG using docking was smaller (1.3 kcal/mol [lysine binds 8-fold more tightly]), which is qualitatively consistent with the experimental results (Fig. 3B, blue circle). This agreement between experimental and docking calculations supports that additional docking calculations might identify variants with less sensitivity to inhibition by lysine. Interestingly, among all the variants, the Ser385Glu variant showed the highest relative affinity for 2-OG over lysine, with a predicted 1.2-fold preference for 2-OG over lysine (0.1 kcal/mol), mainly due to a higher affinity for 2-OG (Fig. 3A and B, green circle). Unlike in the case of the Phe and Glu variants, the binding affinity of the Ser385Leu variant to 2-OG (−6.1 kcal/mol) was greatly decreased compared with that of the WT (−7.7 kcal/mol) and other variants (Fig. 3A). These *in silico* simulations suggest that the glutamate substitution at Ser385 increases the binding affinity to 2-OG, leading to less sensitivity to lysine feedback inhibition, while the leucine substitution would reduce the affinity to 2-OG.

### Effect of amino acid substitutions at Ser385 on the HCS activity of ScLys20.

**FIG 4:**
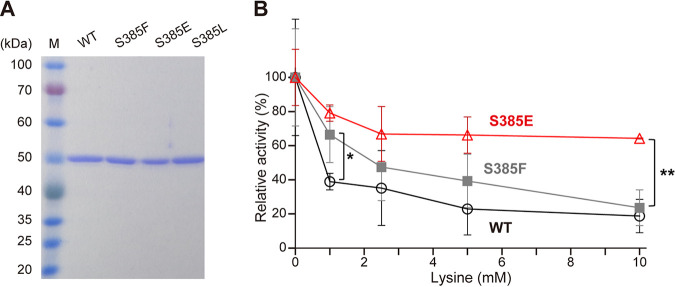
Effects of lysine on HCS activity. (A) SDS-PAGE of the purified recombinant HCSs. Lane M, molecular mass standards; WT, S385F, S385E, and S385L, wild type and S385F, S385E, and S385L mutant variants of the recombinant ScLys20. (B) Effect of lysine on HCS activity. The HCS activities of the wild type (open circles) and S385F (filled gray squares) and S385E (open red triangles) ScLys20 variants were measured in the presence of lysine. The relative activities are expressed corresponding to the parameters in the absence of lysine. The values are means and standard deviations of results from three independent experiments. Asterisks indicate statistically significant differences between two enzymes (Student’s *t* test): *, *P* < 0.05; **, *P* < 0.01.

To confirm the results of *in silico* docking, we purified the recombinant WT-ScLys20 and S385F-, S385E-, and S385L-ScLys20 variants from Escherichia coli cells (Fig. 4A) and measured their HCS activities (Table 1). There was no significant difference in the apparent *K_m_* values for AcCoA among the WT and S385F and S385E variants, but the *k*_cat_/*K*_m_ value for AcCoA of the S385F and S385E variants was slightly increased compared with that of WT. On the other hand, the apparent *K_m_* values for 2-OG of the S385F (1.99 mM) and S385E (3.11 mM) variants were improved compared with that of the WT-ScLys20 (4.60 mM), whereas that of the S385L variant was exacerbated to 9.50 mM. These results were almost consistent with the *in silico* estimation that the binding affinities to 2-OG of the S385F and S385E variants increased and that of the S385L variant decreased. In contrast to the *in silico* estimation, the S385F variant displayed higher affinity to 2-OG than the S385E variant. The difference between *in silico* estimation and experimental results suggests there were other unknown factors that were not included in the calculations, but affected the binding to 2-OG. The substitution with phenylalanine and glutamate at Ser385 also improved the *k*_cat_ and *k*_cat_/*K_m_* values for 2-OG compared with those of the WT.

**TABLE 1:** Kinetic parameters of ScLys20

Lys20	2-OG^*a*^			AcCoA^*a*^		
	*K_m_* (mM)	*k*_cat_ (s^−1^)	*k*_cat_/*K_m_* (mM^−1^·s^−1^)	*K_m_* (μM)	*k*_cat_ (s^−1^)	*k*_cat_/*K_m_* (μM^−1^·s^−1^)
WT	4.60 ± 1.26	0.287 ± 0.028	0.0625	24.2 ± 4.21	0.269 ± 0.014	0.0111

Variant						
S385F	1.99 ± 0.45	0.483 ± 0.031	0.243	22.8 ± 4.88	0.373 ± 0.023	0.0164
S385E	3.11 ± 0.53	0.468 ± 0.026	0.150	16.1 ± 1.62	0.241 ± 0.0065	0.0149
S385L	9.50 ± 1.99	0.436 ± 0.041	0.046	11.5 ± 2.16	0.114 ± 0.0099	0.00992

a The values shown are means and standard deviations of results from three independent experiments.

The HCS activity of WT-ScLys20 was markedly inhibited and that of the S385F variant was higher than that of WT-ScLys20 in the presence of 1 mM lysine, in agreement with previous results (26). However, the remaining activity of the S385F variant was gradually decreased with an increase in lysine concentration and was almost the same as that of WT-ScLys20 in the presence of 10 mM lysine (Fig. 4B). On the other hand, the relative activity of the S385E variant was 64%, even in the presence of 10 mM lysine, which was much higher than that of the S385F variant (23%). The level of activity in the S385E variant was still 13%, even in the presence of 50 mM lysine (data not shown). The half-maximal inhibitory concentration (IC_50_) values of the HCS enzymes were determined as 1.0 ± 0.14 mM for the WT, 2.0 ± 0.68 mM for the S385F variant, and 3.2 ± 0.61 mM for the S385E variant. The higher IC_50_ value of the S385E variant than those of WT and the S385F variant indicates that the serine-to-glutamate substitution at position 385 (S385E) conferred a much higher level of insensitivity to the lysine feedback inhibition than S385F, as expected from the *in silico* estimation.

**FIG 5:**
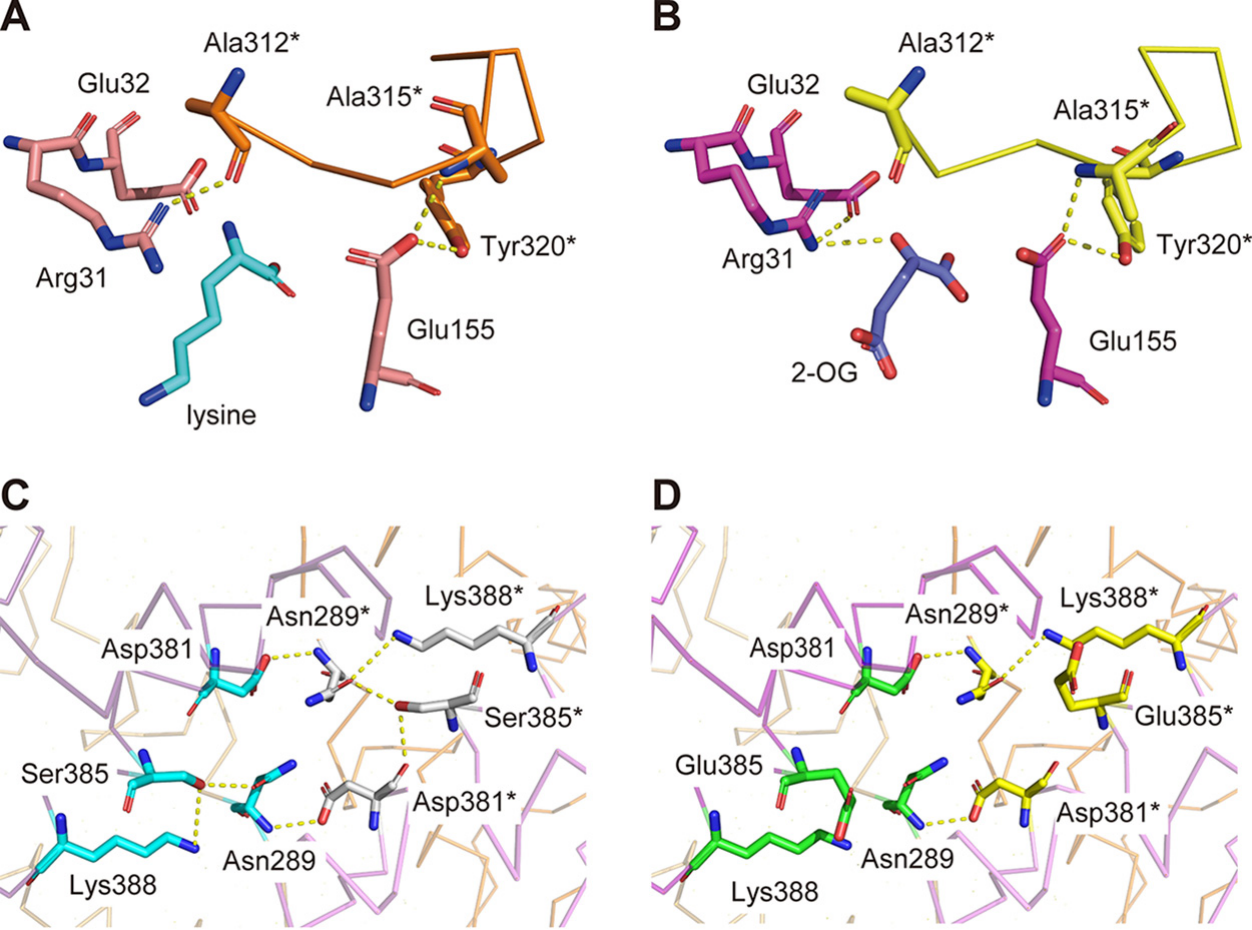
Comparison of the interaction of the TIM barrel domain with the subdomain between lysine and the 2-OG complex structure of ScLys20 and predicted changes in subdomain II by the glutamate substitution at Ser385. (A) Local structure around the ligand binding site of ScLys20 homology model (lysine complex). The inhibitor lysine, Arg31, Glu33, and Glu155 in the active center, and Ala312*, Ala315*, and Tyr320* in C-terminal subdomain II (the asterisk indicates residues from another subunit in the homodimer) are shown in a stick model in cyan, pink, and orange, respectively. Residues between Ala312* and Tyr320* are shown in a ribbon model. The expected hydrogen bonds are shown as yellow dots. (B) Local structure around the ligand binding site of the ScLys20 homology model (2-OG complex). A homology model of ScLys20 bound with 2-OG was constructed using SWISS-MODEL, with SpHCS 2-OG complex (PDB no. 3IVU) as a template. Substrate 2-OG, Arg31, Glu33, and Glu155, and Ala312*, Ala315*, and Tyr320* are shown in a stick model in blue, magenta, and yellow, respectively. (C) Interactions within Ser385, Asn289, Asp381, and Lys388 intra- and intermonomers of WT ScLys20. Backbones of the C-terminal subdomains are shown in a ribbon model. Ser385, Asn289, Asp381, and Lys388 in chain A are shown in a stick model in cyan color, and those in chain B (indicated via asterisks) are in white. The hydroxyl group in the side chain of Ser385 is predicted to form hydrogen bonds with Asn289, Asp381, and Lys388. Asn289-Asp381* and Asn289*-Asp381* interactions were also observed. (D) Interactions within Glu385, Asn289, Asp381, and Lys388 intra- and intermonomers of the S385E variant. Glu385, Asn289, Asp381, and Lys388 in chain A are shown in a stick model in green, and those in chain B (indicated via asterisks) are in yellow. The hydrogen bonds within Ser385 to Asn289, Asp381, and Lys388 predicted in the WT ScLys20 were not observed with the glutamate substitution.

Structural analysis of HCSs demonstrated that the C-terminal subdomain is important for recognition of the inhibitor lysine. Thus, we compared the ScHCS homology model with the crystal structures of SpHCS and TtHCS to elucidate the effect of the glutamate substitution at Ser385 of ScHCS on sensitivity to lysine inhibition. In the case of TtHCS, His292 and Tyr303 are located near the active center when 2-OG binds, whereas Tyr297 is present in the vicinity of lysine and stabilizes the enzyme-ligand complex when lysine binds (21). On the other hand, the crystal structure of SpHCS suggests that the C-terminal subdomain of fungal HCSs is involved in lysine recognition in a different manner. In the apo and lysine complex structure of SpHCS, the guanidinium group of Arg43 in the active center created a hydrogen bond with the carbonyl group of Ala324* (the asterisk indicates residues from another subunit in the homodimer). In contrast, the conformational change in the side chain of Arg43 and an interaction between its guanidinium group and 2-OG were observed in the 2-OG complex (19). These arginine and alanine residues are also conserved in ScHCS (Arg31 and Ala312, respectively). In the homology model of ScLys20, a hydrogen bond between Arg31 and Ala312* is observed in the lysine complex, not in the 2-OG complex, due to the conformational change in Arg31 that allows Arg31 to interact with 2-OG in the same manner as SpHCS (Fig. 5A and B). These conformational changes between the substrate and inhibitor complex suggest the interaction between Arg31 and Ala312* would contribute to stabilization of the lysine complex. Ser385 is located far from the active center TIM barrel and Ala312 in the same monomer, suggesting that Ser385 does not directly interact with ligands or residues in the active center. However, the hydroxyl group of Ser385 is predicted to form multiple hydrogen bonds with the amino group in the side chain of Lys388, the carboxamide group in the side chain of Asn289, and the carbonyl group in the main chain of Asp381 (Fig. 5C). Furthermore, interactions between the amino group in the main chain of Asn289 and the carboxyl group in the side chain of Asp381* (also between Asp381 and Asn289*) are predicted, suggesting that these interactions contribute to maintenance of the homodimer structure. The substitution of Ser385 with glutamate would disrupt these hydrogen bond networks within Asn289, Asp381, and Lys388 (Fig. 5D). This may cause a conformational change in the C-terminal subdomain, thereby altering the equilibrium between the lysine and 2-OG complex of ScHCS: for instance, the conformational change caused by amino acid substitution breaks the interaction between Ala312* and Arg34, even in the lysine complex, leading to instability of the lysine complex.

### Effect of amino acid substitutions at Ser385 on lysine productivity of ScLys20.

**FIG 6:**
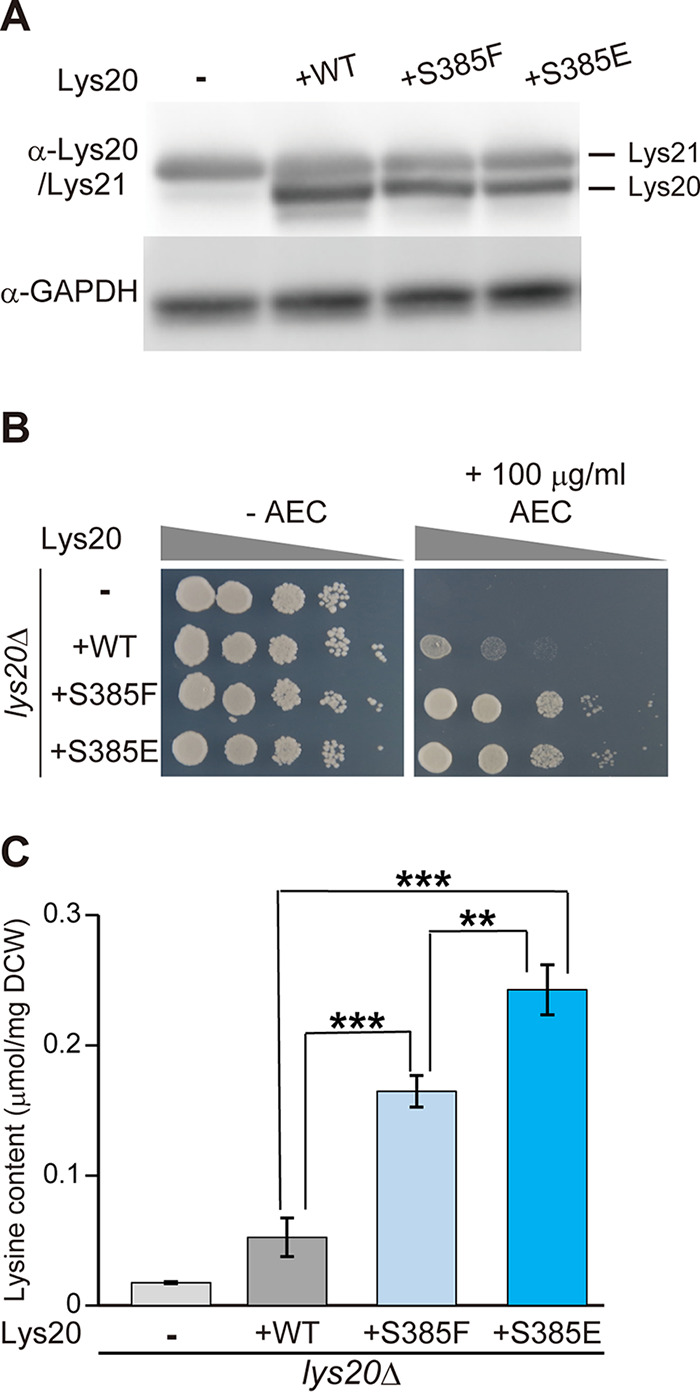
Effects of the Ser385 variants on yeast cells. X2180-1A *lys20*Δ strains harboring pYC150 empty vector, pYC150_*LYS20*, pYC150_*LYS20^S385F^*, and pYC150_*LYS20^S385E^* are represented by “−,” “+WT,” “+S385F,” and “+S385E,” respectively. (A) Protein levels of Lys20 and Lys21 in yeast cells. The GAPDH level is shown as a protein loading control in Western blotting. (B) AEC resistance of yeast cells expressing the WT and Ser385 variants of ScLys20. Optical densities at 600 nm of each transformant were serially diluted to 10^1^- to 10^4^-fold (from left to right) and applied as spots onto SD-N+Alla plates containing nourseothricin (left panel) and SD-N+Alla plates containing nourseothricin plus 100 μg/ml of AEC (right panel) agar medium, and the plates were incubated at 30°C for 2 days. (C) Intracellular lysine content of yeast cells expressing the WT and Ser385 variants of ScLys20 (μmol per mg dry cell weight [DCW]). X2180-1A *lys20*Δ expressing the WT and Ser385 variants of *LYS20* genes were cultured SD-N+Alla liquid medium containing nourseothricin at 30°C for 72 h. The values are means and standard deviations of results from three independent experiments. Asterisks indicate statistically significant differences between two strains (Student’s *t* test): **, *P* < 0.01; ***, *P* < 0.001.

The *in vitro* results suggest that expression of the S385E variant increases intracellular lysine content compared with that in the S385F variant-expressing cells. To analyze the effect of amino acid substitutions at Ser385 on lysine productivity, the *lys20*Δ strain was constructed from the WT strain (X2180-1A), and the WT and mutant *LYS20* genes encoding the S385F and S385E variants were expressed by their own promoters and terminators in the *lys20*Δ strain. There was no significant difference in the growth rates of transformants, and they reached the stationary phase after 72 h of cultivation (data not shown). Western blotting of total proteins in the stationary phase of yeast cells showed that the protein levels of ScLys20 and ScLys21 were almost the same among yeast cells expressing the WT-, S385F-, and S385E-ScLys20 (Fig. 6A). To evaluate the effects of the expression of the ScLys20 variants on AEC sensitivity, the growth phenotypes of *lys20*Δ cells expressing ScLys20 were compared in the presence of AEC on SD-N+Alla agar plates, as described below. As shown in Fig. 6B, the *lys20*Δ strain was highly sensitive to AEC, and the expression of WT-ScLys20 partially recovered the growth defect caused by AEC. In contrast, the expression of the S385F and S385E variants increased the resistance to AEC, suggesting that these variants enhanced the lysine productivity in yeast cells (Fig. 6B). Next, we determined the cellular amino acid levels of the *lys20*Δ-derived transformants (Fig. 6C). A small amount of lysine was detected in the case of *lys20*Δ cells harboring the empty vector, due to the presence of ScLys21. The S385F variant ScLys20 showed a prominent 3.1-fold increase in intracellular lysine content compared with the WT enzyme. Interestingly, the lysine contents in *lys20*Δ cells expressing the S385E variant were 4.6- and 1.5-fold higher than those in *lys20*Δ cells expressing WT- and S385F-ScLys20, respectively. These results indicate that the extreme feedback inhibition desensitization of the S385E variant leads to intracellular lysine accumulation and that the molecular design of HCS for removal of feedback inhibition will greatly contribute to the construction of lysine-overproducing yeasts. Rational design has been used to enhance the high-temperature stability and to change the enantioselectivity of enzymes (28, 29). Our results indicate that *in silico* design can be adapted for removal of the feedback inhibition and thus has great potential as a means of efficiently obtaining variant enzymes with improved function, rather than isolation by random mutagenesis.

Yeast cells expressing the S385E variant will be expected to accumulate approximately 1 mmol of lysine in the cells harvested from a 1-liter cultivation; however, further improvement of the lysine productivity may be necessary for industrial utilization. A dramatic increase in lysine productivity in S. cerevisiae could be achieved both by the optimization of other amino acid residues responsible for the lysine feedback inhibition, such as Arg276, and by the constitutive activation of the genes involved in the AAA pathway by engineering of the transcriptional regulator SpLys14 (33). The biosynthesis of lysine requires a large amount of the reduced form of nicotinamide adenine dinucleotide phosphate (NADPH), and thus an improvement of the NADPH regeneration system may also be needed (34). The expected lysine titer (1 mmol in the cells from a 1-liter culture) of yeast cells expressing the S385E variant is much lower than that of C. glutamicum (over 40 mM in culture broth) (34). However, most of the lysine is secreted to culture medium in C. glutamicum, while the yeast S. cerevisiae accumulates lysine in the cell. Thus, overproduction of lysine in yeast cells is expected to be utilized in a different way from that in C. glutamicum, such as yeast extract with high lysine content.

### Conclusion.

The present study demonstrated the use of *in silico* docking simulation for rational engineering of the yeast HCS that is desensitized to lysine feedback inhibition and thus promotes intracellular accumulation of lysine. The approach described here indicates that the combination of *in silico* simulation and experimental validation can provide an efficient method for the design of key enzymes to enhance the productivity of target compounds.

## MATERIALS AND METHODS

### Strains and media.

We used S. cerevisiae wild-type (WT) strain X2180-1A (*MAT*α *SUC2 mal mel gal2 CUP1*). The *LYS20* gene of X2180-1A was disrupted, and the resulting *lys20*Δ strain was used as a host strain for expression of the WT and variants of ScLys20. Disruption of the *LYS20* gene was conducted as follows. The deletion cassette for the *LYS20* allele was amplified from the genomic DNA of the S. cerevisiae *lys20*Δ strain with the BY4741 background (*MAT*α *his3*Δ*1 leu2*Δ*0 met15*Δ*0 ura3*Δ*0*) (obtained from the yeast single deletion library at Euroscarf) with primers LYS20_BamHI_Fw and LYS20_SacI_Rv (Table 2). The amplified DNA fragment, including P*_LYS20_*-KanMX4-T*_LYS20_* (*LYS20* open reading frame [ORF] replaced by KanMX4) was introduced into strain X2180-1A, and the *LYS20*-disrupted strain was then selected by G418 resistance. Escherichia coli strains DH5α [F^−^ λ^−^ ϕ80*lacZ*ΔM15 Δ(*lacZYA argF*)*U169 deoR recA1 endA1 hsdR17*(*r_K_*^−^*m_K_*^+^) *supE44 thi-1 gyrA96*] and BL21(DE3) [F^–^ *ompT hsdS*(r_B_^–^ m_B_^–^) *gal dcm* λ(DE3) (*lacI lacUV5-*T7 gene 1 *ind1 sam7 nin5*)] were used for construction of expression plasmids and for expression of the recombinant ScLys20, respectively. Yeast transformants were cultivated in a synthetic minimal medium SD-N+Alla (2% glucose, 0.67% yeast nitrogen base without amino acids and ammonium sulfate, and 0.5% allantoin as a nitrogen source) containing 200 μg/ml nourseothricin, unless otherwise stated. E. coli strains were cultured in Luria-Bertani (LB) medium (0.5% yeast extract, 1% tryptone, and 1% NaCl) containing appropriate antibiotics or in M9CA medium (0.4% glucose, 2% Casamino Acids, 65 mM sodium/potassium phosphate, 8.6 mM NaCl, 18.7 mM ammonium chloride, and 1 mM MgSO_4_) containing 100 μg/ml ampicillin.

**TABLE 2:** Primers used in this study

Primer	Sequence (5′→3′)^*a*^
Gene cloning	
LYS20_gateway_Fw	GGGGACAAGTTTGTACAAAAAAGCAGGCTTAATGACTGCTGCTAAACCAAATCC
LYS20_gateway_Rv	GGGGACCACTTTGTACAAGAAAGCTGGGTGTTAGGCGGATGGCTTAGTCCGC

Gene cloning and disruption of *LYS20* allele	
LYS20_BamHI_Fw	ACTGGATCCGTATACTGCGTGCGCTTGAGATTC
LYS20_SacI_Rv	CTGGAGCTCGGACGAACTTTGCGCGAAGTGG

Site-directed mutagenesis	
lys20_S385F_Fw	CGATGATGTTGACTT*TATCATCAAGAACTTCCACGCAGAG
lys20_S385F_Rv	CTCTGCGTGGAAGTTCTTGATGATAA*AGTCAACAT
lys20_S385E_Fw	ATCGATGATGTTGACG*A*A*ATCATCAAGAACTTC
lys20_S385E_Rv	GAAGTTCTTGATGATT*T*C*GTCAACATCATCGAT
lys20_S385L_Fw	ATCGATGATGTTGACT*T*A*ATCATCAAGAACTTC
lys20_S385L_Rv	GAAGTTCTTGATGATT*A*A*GTCAACATCATCGAT

a The underlined nucleotides indicate BamHI and SacI restriction sites. The asterisks indicate the positions of the nucleotide mutation.

### Docking simulation of the WT and its ScLys20 variants with ligands.

Docking used a homology model of the cytostolic homocitrate synthase from S. cerevisiae (ScLys20 [UniProtKB accession no. P48570]), which was downloaded from the SWISS-MODEL repository (31). The template protein used for construction of the model was the mitochondrial cytosolic homocitrate synthase from S. pombe (SpHCS; 78% sequence identity to ScLys20 [PDB ID no. 3MI3]). The model is a homodimer containing the bound lysine, which was removed for the docking calculations. The model matches the template protein closely, except the region near residue 120.

The Ser385 variants were constructed using PyMOL (the PyMOL Molecular Graphics System version 2.4; Schrödinger, LLC) using the lowest-energy rotamer in each case. Docking was carried out using SwissDock (32) using default parameters. The docking calculation returned approximately 35 clusters of ligand-protein orientations. Each cluster contained up to eight similar poses. Some clusters contained the ligand bound within one of the active sites of the dimer, while other clusters contained the ligand bound on the protein surface and were ignored. The binding energies of each pose within the clusters placing the ligand in the active site were averaged, and the standard deviation of these energies was calculated.

### Construction of expression plasmids for the *LYS20* genes.

To construct plasmids for expression of the recombinant proteins, the *LYS20* gene was amplified from the genomic DNA of S. cerevisiae X2180-1A by PCR with the primers LYS20_gateway_Fw and LYS20_gateway_Rv (Table 2). The PCR-amplified DNA fragment was introduced into the pDONR221 vector (Thermo Scientific) using BP Clonase II (Thermo Scientific), resulting in pDONR221_*LYS20*. The point mutations were introduced into the *LYS20* gene on pDONR221 with the primers listed in Table 2, leading to the S385F, S385E, and S385L substitutions on ScLys20. The nucleotide sequences of the *LYS20* genes were verified and they were transferred to the pET53-dest expression vector (Thermo Scientific) using LR Clonase II (Thermo Scientific), resulting in pET53_*LYS20*, pET53_*LYS20^S385F^*, pET53_*LYS20^S385E^*, and pET53_*LYS20^S385L^*.

The expression plasmids for the WT and ScLys20 variants were constructed as follows. The DNA fragment, including 1,000 bp upstream and downstream of the open reading frame of *LYS20*, was amplified from the genomic DNA of S. cerevisiae X2180-1A with primers LYS20_BamHI_Fw and LYS20_SacI_Rv (Table 2). The amplified DNA was digested with BamHI-SacI and ligated into the same site of the expression vector pYC150 (35), resulting in pYC150_*LYS20*. The point mutations were introduced into the *LYS20* gene on pYC150 as described above, resulting pYC150_*LYS20^S385F^* and pYC150_*LYS20^S385E^*.

### Expression and purification of the N-terminal His-tagged recombinant ScLys20.

E. coli BL21(DE3) cells harboring pET53-*LYS20* (WT), pET53-*LYS20^S385F^*, pET53-*LYS20^S385E^*, and pET53-*LYS20^S385L^* were cultivated in 100 ml of M9CA medium containing ampicillin and grown at 37°C to an optical density 600 nm (OD_600_) of 0.8. The cells were cooled on ice for 5 min, and isopropyl-β-d-1-thiogalactopyranoside (IPTG) was added to a final concentration of 0.2 mM. After 20 h of cultivation at 18°C, the cells were harvested by centrifugation and suspended in 7 ml of buffer A (50 mM HEPES-KOH [pH 7.5] and 300 mM KCl). The cell suspension was homogenized under cooling and then centrifuged to remove insoluble fraction. The supernatant was filtrated through a 0.45-μm-pore filter and subsequently applied onto a nickel affinity column (Ni Sepharose 6 Fast Flow; GE Healthcare Life Sciences). After the column was washed with buffer A containing 80 mM imidazole, the recombinant proteins were eluted by buffer A supplemented with 500 mM imidazole and 10% glycerol.

### Enzymatic activity of ScLys20.

HCS activity was measured by the production of CoA using dichloroindophenol (DCPIP) as previously described, with slight modification (36). The reaction mixture (final volume, 1 ml) contained 100 mM HEPES-KOH (pH 7.5), 75 μM DCPIP, and various concentrations of 2-oxoglutarate (2-OG) and acetyl CoA (AcCoA). The reaction mixture was preequilibrated for 3 min at 30°C, and then the reaction was initiated by the addition of 8 μg of purified ScLys20. HCS-dependent degradation of DCPIP was monitored at 595 nm with a DU-800 spectrophotometer (Beckman Coulter) and maintained at 30°C. For steady-state kinetics, when the concentration of 2-OG was kept at 10 mM, the concentrations of AcCoA were varied (5 to 250 μM). With a fixed concentration of 100 μM AcCoA, the concentration of 2-OG was 1 to 25 mM. In order to examine the feedback inhibition sensitivity of HCSs, the concentrations of 2-OG and AcCoA were fixed at 10 mM and 100 μM, respectively, and lysine was added to the reaction mixture at a concentration of 0 to 10 mM. For determination of the 50% inhibitory concentration (IC_50_) values of the HCS enzymes, the concentrations of 2-OG and AcCoA were fixed at 2 mM and 100 μM, respectively, and lysine was added to the reaction mixture at a concentration of 0 to 10 mM. The reaction rate was calculated with the extinction coefficient of DCPIP, 1,920 M^−1^·cm^−1^. One unit of activity was defined as the amount of enzyme required to produce 1 μmol of CoA per min. Kinetic parameters of each enzyme were calculated with GraphPad Prism version 7 (GraphPad Software) using nonlinear regression analysis.

### AEC sensitivity of yeast cells expressing ScLys20.

The S. cerevisiae *lys20*Δ strain harboring pYC150_*LYS20*, pYC150_*LYS20^S385F^*, and pYC150_*LYS20^S385E^* was precultured for 2 days at 30°C, inoculated into a new medium, and grown to an OD_600_ of 1.0. Yeast cells were collected and washed with sterilized water two times. Serially diluted yeast cells were applied as spots onto SD-N+Alla plates containing 200 μg/ml nourseothricin and 100 μg/ml *S*-aminoethyl-cysteine (AEC) and incubated for 2 days at 30°C.

### Lysine content of yeast cells expressing ScLys20.

S. cerevisiae transformants expressing the WT and ScLys20 variants were precultured for 2 days at 30°C and then inoculated into the same medium at an OD_600_ of 0.1. After cultivation for 72 h at 30°C, yeast cells were collected by centrifugation and then washed twice with sterilized water. Harvested cells were resuspended in sterilized water, and the suspension was adjusted to an OD_600_ of 20. Consequently, intracellular amino acids in an aliquot (0.5 ml) of the cell suspension were extracted by boiling water at 100°C for 20 min. After centrifugation, each supernatant was subsequently quantified with an amino acid analyzer by ion-exchange chromatography and postcolumn ninhydrin derivatization (JLC-500/V2; JEOL). The content of each amino acid was represented as μmol per mg dry cell weight (DCW).

### Western blot analysis.

For the detection of expression levels of ScLys20 and ScLys21 in the *lys20*Δ strain expressing ScLys20, yeast cells were cultivated for 72 h under the same conditions described above. Harvested cells were suspended in 100 mM NaOH and incubated for 10 min at room temperature. Proteins in the whole-cell extracts were separated by SDS-PAGE (10% polyacrylamide), transferred to a polyvinylidene difluoride membrane, blocked with Blocking One (Nacalai Tesque) in Tris-buffered saline with Tween 20 (TBST) at room temperature for 60 min, and reacted with an anti-Lys20/Lys21 mouse antibody (31F5; Novus Biologicals) in Can Get Signal Immunoreaction Enhancer Solution 1 (Toyobo) at 1:50,000 dilutions overnight. As a protein-loading control, an anti-glyceraldehyde-3-phosphate dehydrogenase (GAPDH) rabbit antibody (Nordic Immunological Laboratories) in Can Get Signal Immunoreaction Enhancer Solution 1 (Toyobo) at 1:10,000 dilutions was used as a primary antibody overnight. After several washing steps with TBST, the membrane was incubated for 60 min with horseradish peroxidase-conjugated anti-mouse IgG (Promega) in Can Get Signal Immunoreaction Enhancer Solution 2 (Toyobo) at 1:2,000 dilutions as a secondary antibody. After several washing steps with TBST, the target proteins were visualized by the Amersham ECL Prime Western blotting detection reagent (GE Healthcare) and detected using a Fuji LAS4000 imager (GE Healthcare).

### Data availability.

The data underlying this article are available in the article.
